# Evaluation of a Pretargeting Strategy for Molecular Imaging of the Prostate Stem Cell Antigen with a Single Chain Antibody

**DOI:** 10.1038/s41598-018-22179-y

**Published:** 2018-02-28

**Authors:** Lena Tienken, Natascha Drude, Isabell Schau, Oliver H. Winz, Achim Temme, Elmar Weinhold, Felix M. Mottaghy, Agnieszka Morgenroth

**Affiliations:** 1 Department of Nuclear Medicine, University Hospital RWTH Aachen, 52074 Aachen, Germany; 2 Department of Neurosurgery, Experimental Neurosurgery/Tumor Immunology, TU Dresden, 01307 Dresden, Germany; 3 German Cancer Consortium (DKTK), partner site Dresden, German Cancer Research Center (DKFZ), Heidelberg, Germany; 4 Institute of Organic Chemistry, RWTH Aachen University, 52074 Aachen, Germany; 5 Department of Radiology and Nuclear Medicine, MUMC+, Maastricht, The Netherlands

## Abstract

In pretargeted radio-immunotherapy, the gradual administration of a non-radioactive tumor antigen-addressing antibody-construct and the subsequent application of a radioactive labeled, low molecular weight substance enable a highly effective and selective targeting of tumor tissue. We evaluated this concept in prostate stem cell antigen (PSCA)-positive cancers using the antigen-specific, biotinylated single chain antibody scFv(AM1)-P-BAP conjugated with tetrameric neutravidin. To visualize the systemic biodistribution, a radiolabeled biotin was injected to interact with scFv(AM1)-P-BAP/neutravidin conjugate. Biotin derivatives conjugated with different chelators for complexation of radioactive metal ions and a polyethylene glycol linker (n = 45) were successfully synthesized and evaluated *in vitro* and in a mouse xenograft model. *In vivo*, the scFv(AM1)-P-BAP showed highly PSCA-specific tumor retention with a PSCA^+^ tumor/PSCA^-^ tumor accumulation ratio of ten. PEGylation of radiolabeled biotin resulted in lower liver uptake improving the tumor to background ratio.

## Introduction

Monoclonal antibodies (mAb) are suitable to target tumor associated antigens due to their high binding affinitiy^1^. In radio-immunotherapy (RIT) radioactive isotopes are conjugated to antibodies for selective detection and endogenous radiation of target tissue while sparing non-target tissue. Depending on the used isotope the antibodies can be employed for diagnostic imaging with positron emission tomography (PET) or single photon computed tomography (SPECT) or therapeutic approaches^2^. The bivalent binding capacity increases the antigen affinity and confers long retention at the binding site which on the other hand limits the intratumoral biodistribution of an antibody (“binding site barrier”)^3^. Moreover, the high molecular weight leads to long serum half-life, hence results in a poor contrast in imaging applications (PET and SPECT)^4^ and apparently limited tumor penetration^5^. An additional challenge with such antibody conjugates is the inherent interaction of the fragment crystallizable antibody region (Fc region) with Fc receptors expressed on certain cells of the immune system. After binding, the antibody is recycled as a part of the protective function of the immune system. To circumvent these limitations different antibody constructs with lower molecular weights and without a Fc region were developed. Single chain variable fragments (scFv) with a molecular weight between 27 and 30 kDa are known as one of the smallest “antibody formats”^6^. It consists of variable light and heavy domains of an antibody which are typically fused by a peptide-linker containing 10–25 Glycine and Serine amino acids^7^. Thus, scFv contain the complete monovalent antigen binding site preserving the binding specificity of the parental antibody^8^. Even though monovalency results in a lower binding affinity, scFv show better tumor penetration and preferable pharmacokinetics. The missing Fc region results in a reduced elimination by B-lymphocytes and macrophages. Still the fast blood clearance and short biological half-life (t_1/2_ = 0.5–2 h)^9^ of scFv might limit its utility as radiopharmaceuticals at least for therapeutic application. To minimize or avoid this first pass elimination effect scFv can be structurally modified^10^. Modifications such as glycosylation sites, additional linkers or functional domains can result in optimized molecular weight, hydrophilicity and/or prevention of protein aggregation^11^.

**Figure 1:**
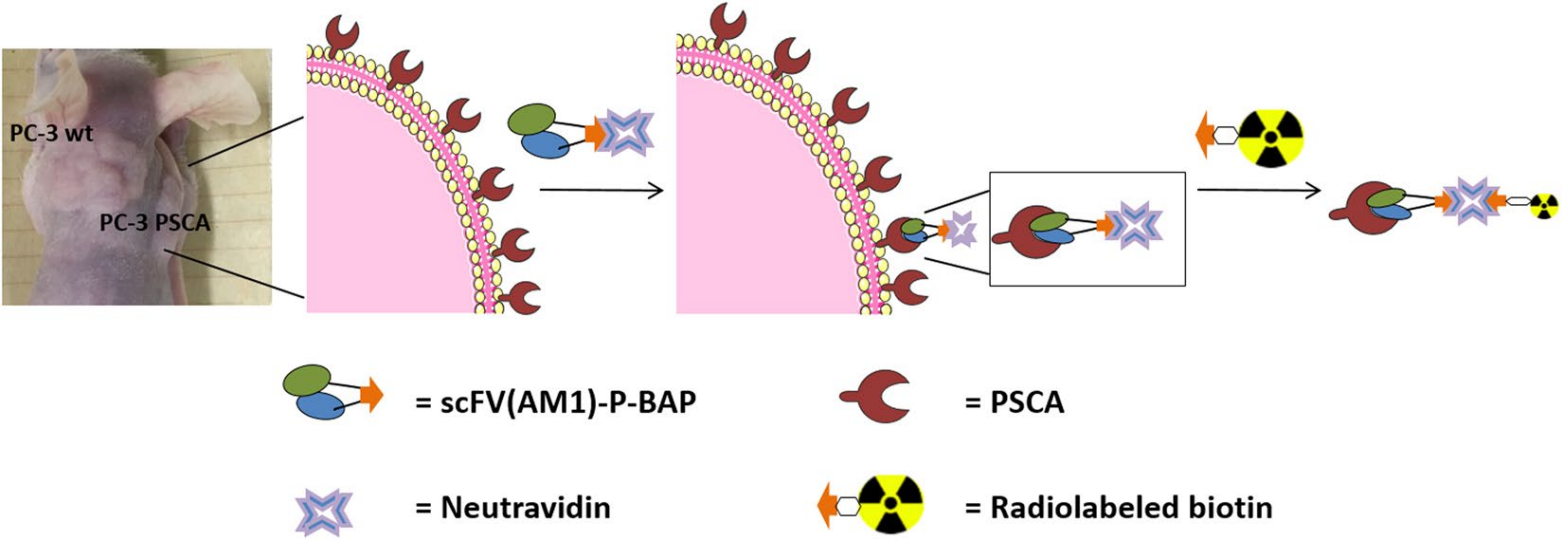
Schematic representation of the evaluated pretargeting strategy.

For radio-immunotherapy, the radiation dose is related to the circulation time of the radiolabeled antibody construct in blood and the resulting exposure of target and non-target tissue. With the application of so-called pretargeting strategies, the limiting, systemic toxicity can be reduced significantly. Conceptually, in pretargeted radio-immunotherapy the non-radioactive antibody construct is injected first followed by subsequent injection of the radiolabeled, low-molecular substrate. The specific and high tumor selectivity of the antibody leads to an efficient targeting of tumor and minimizes radiation exposure of healthy tissue^12^. This is due to rapid binding of the corresponding radioactive substrate to the antibody construct while the un-bound radioactivity is quickly eliminated renally^13^. Consequently, when compared to directly labeled macromolecules, pretargeting enables administration of high doses of radioactivity without increasing systemic toxicity^14^. The high accumulation of the antibody construct in the tumor tissue as well as the fast clearance of the unbound low-molecular weight substrate finally results in a high tumor to background ratio^15^. Figure 1 illustrates the herein evaluated strategy.

Different pretargeting approaches exist which are based on (neutr)avidin/biotin systems, bispecific antibodies and oligonucleotide/antisense oligonucleotide analogs^16^. All of these receptors/effectors systems are distinguished by high binding constants and fast binding kinetics^17^. The avidin/biotin system has one of the strongest known non-covalent interactions. The dissociation constant K_D_ between one of the four identical subunits of the tetramer and the low-molecular weight biotin is about 10–15 mol/L^18^. However, the high isoelectric point of avidin (pI = 10.5) results in non-specific binding therefore streptavidin is preferred in biochemical applications such as pretargeting strategies^19^. Streptavidin is a bacterial non-glycosylated tetrameric protein characterized by comparable biotin-binding properties as avidin. Its pI is nearly neutral (pI = 5–6) and has therefore lower tendency towards non-specific interactions^20^. Nevertheless, streptavidin is not entirely free of non-specific binding, exemplified by its motif Arg-Tyr-Asp, similar to Arg-Gly-Asp, the universal recognition site in fibronectin and other adhesion molecules^21^. Furthermore, streptavidin showed a high sustained uptake in the kidneys in former applications of pretargeting strategies^15^. To address these concerns we used neutravidin in our pretargeting approach. The chemical modifications of neutravidin reduced the pI (pI = 6.3) and hence its non-specific binding^22^ while maintaining the high biotin-binding affinity^23^. Due to the application of neutravidin instead of streptavidin, the accumulation in the kidneys as one dose-limiting organ^24^ should efficiently be reduced. A pretargeted radioimmuno-strategy requires a complementary effector molecule like biotin to be conjugated with a chelator to enable labeling with radioactive metal ions. For *in vivo* application, this conjugation has to be stable especially against degradation by biotinidase, an enzyme which recycles biotin from endogenous biocytin^25^. Thus, ordinary synthesis of biotin derivatives resistant to degradation by the endogenous biotinidase is highly desirable. Besides renal accumulation a high uptake in the liver was observed in first clinical trials of pretargeting approaches^26^. The hepatic accumulation of the conjugates can be explained by non-specific uptake in Kupffer cells. Kupffer cells are macrophages residing in the lumen of the liver capillary vessels. They constitute 80–90% of the tissue macrophages present in the body^27^. As a result, a high concentration of conjugates can be detected in the liver. To address this limiting hepatic accumulation we compare two biotin chelator derivatives one with and one without PEG (n = 45) linker. Importantly, PEGylation was shown to prolong the serum half-live which beneficially impacts the general biodistribution^28^.

In this study we address the prostate stem cell antigen (PSCA). PSCA is a glycosyl-phosphatidyl-inositol (GPI) cell surface antigen, which is overexpressed on different tumor entities like prostate, bladder and pancreatic cancer and only marginally expressed, in the corresponding normal tissue^29^. The expression of PSCA correlates with the Gleason Score, the pathological tumor stage and the progression to androgen-independence^30,31^. In addition prostate cancer metastases in bone marrow, lymph node and liver are stained positively for PSCA^32^. This makes PSCA an attractive biological target for an antibody-based therapy. The scFv(AM1) was shown to be a highly selective and effective PSCA-addressing construct^33^. The estimated K_D_ of scFv(AM1) = 2.3 * 10^−6^ mol/L lies in the lower affinity range^34^. The choice of low affinity antiboday(fragment) is based on the fact that in antibody(fragment) based approaches the moderate affinity is associated with the highest tumor accumulation, whereas high affinity is found to produce the lowest tumor accumulation, due to the “binding site barrier”^35^. As shown by Rudnick *et al*. antibodies with the higher affinity also exhibited a greater degree of internalization^36^. For the pretargeting approach scFv(AM1) was engineered by insertion of biotin acceptor peptide (P-BAP) which ensures a site-specific mono-biotinylation. In this study we evaluate the potential of scFv(AM1)-P-BAP/Neutravidin complex and radiolabeled biotin for molecular imaging of PSCA expressing tumors.

## Results and Discussion

For evaluation of a pretargeting strategy with a single chain antibody specific for PSCA-positive tumor cells the scFv(AM1)-P-BAP was generated as described earlier^37^. The C-terminal insertion of P-BAP enabled production of a mono-biotinylated scFv(AM1)-P-BAP and subsequent conjugation to neutravidin at defined stoichiometry. The scFv(AM1)-P-BAP/neutravidin ratio was optimized to ensure protein binding to only one of the four binding sites for biotin. For radiolabeling, biotin was covalently bound to 1, 4, 7-triazacyclononane-N, N′, N″-triacetic acid (NOTA), a chelator that coordinates the positron emitter ^68^Ga (half-life t_1/2_ = 67.6 min) at room temperature, or to deferoxamine (DFO) which is an open chain chelator able to coordinate the long-lived ^89^Zr (t_1/2_ = 78.4 h). The pretargeting strategy was evaluated *in vitro* as well as *in vivo* in xenografted mice with human prostate cancer cell lines (PSCA-overexpressing PC-3 PSCA cells and PC-3 wildtype cells as negative control).

### Synthesis of radioactive biotin conjugates

**Figure 2:**
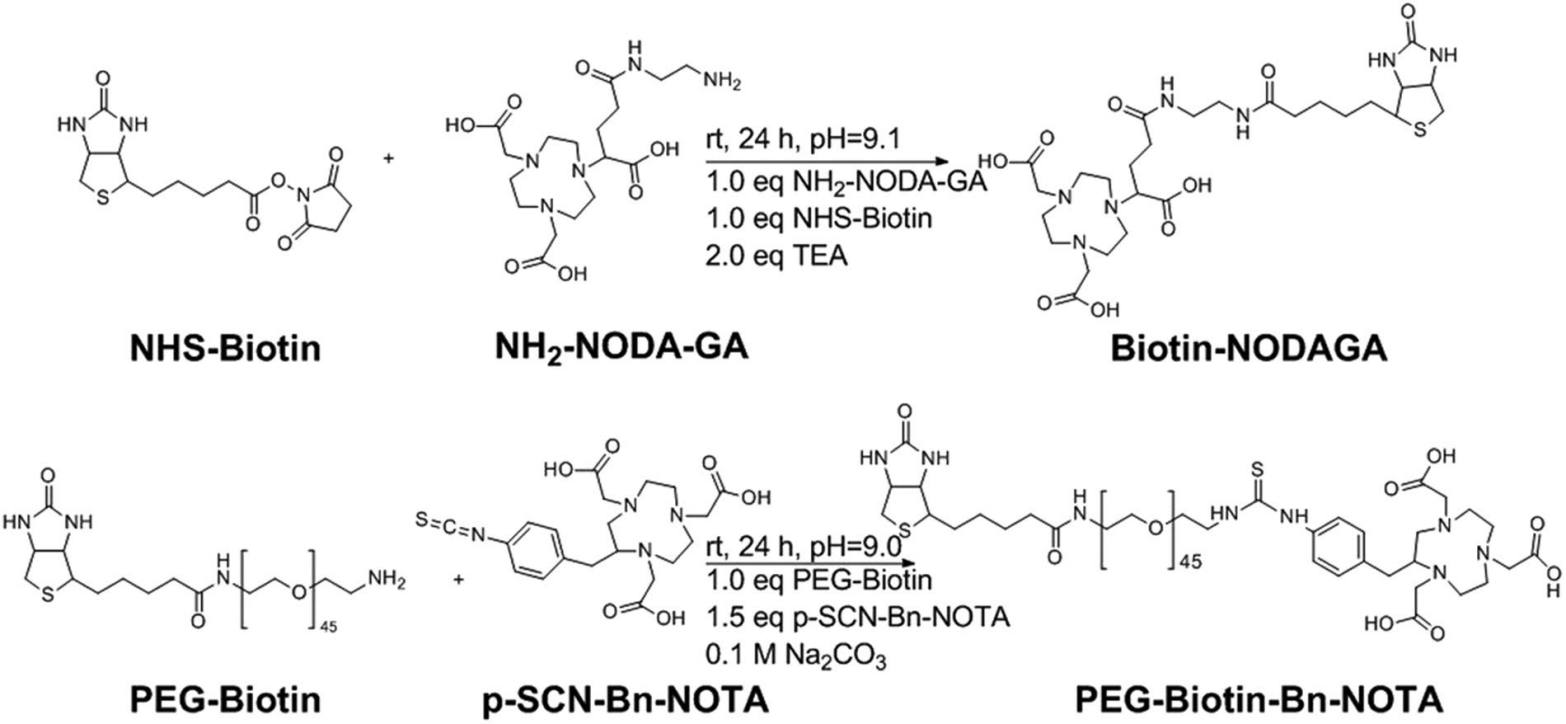
Synthesis of biotin/chelator conjugates.

For an effective pretargeting strategy, synthesis of stable biotin/chelator conjugates is mandatory. In this work, two biotin derivatives with and without a PEG linker (n = 45) were chosen (Fig. 2).

NHS-biotin was reacted with the primary amine of NH_2_-NODA-GA at slightly alkaline pH to form a peptide bond. To introduce a PEG linker, amine functionalized PEG-biotin was reacted with the isothiocyanate group of p-SCN-Bn-NOTA to generate a stable thiourea bond. The impact of PEGylation on binding specificity to PSCA positive cells and biodistribution was evaluated *in vitro* as well as *in vivo*. For quantitative imaging over several days, PEG-Biotin-Bn-DFO was synthesized as described above for PEG-Biotin-Bn-NOTA (SI). The products were analyzed by reversed phase HPLC after radiolabeling with ^68^Ga. Besides UV detection, compounds were detected with a radioactivity detector (SI Figs S2–S4). Furthermore, the products were analyzed by radio-TLC. The biotin/chelator conjugates were obtained with a purity of > 85% (as determined with radio-HPLC). Radiolabeling was performed with a radiochemical yield (rcy) > 90% (as determined with radio-TLC). Products were purified via semipreperative HPLC, lyophilized and redissolved before each experiment. Radiochemical purities (rcp) > 90% were achieved.

### scFv(AM1)-P-BAP/neutravidin ratio

**Figure 3:**
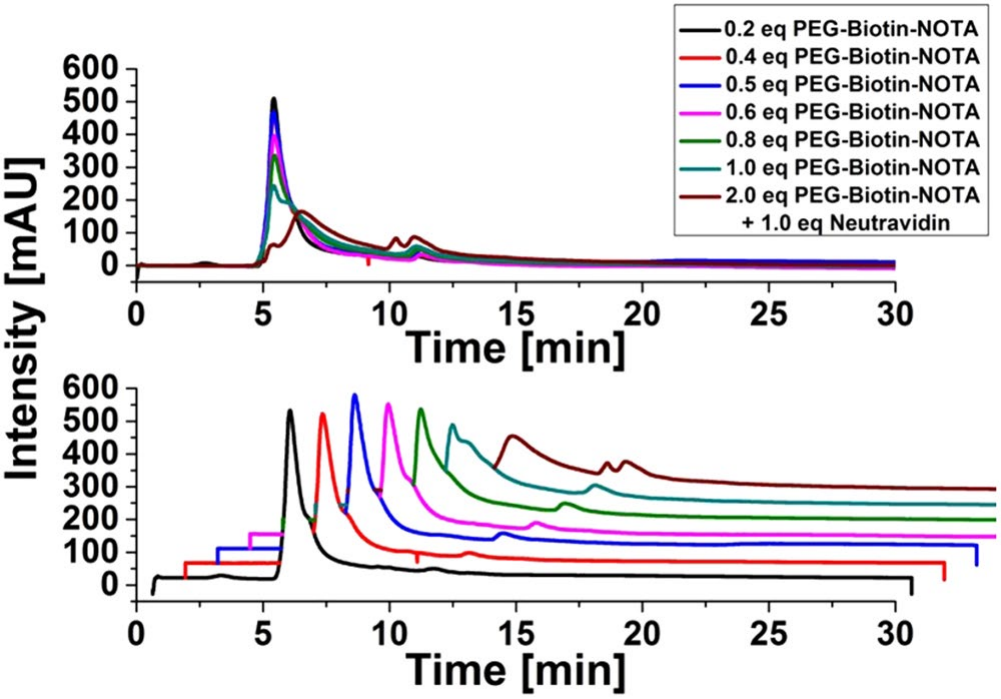
Binding of PEG-biotin-NOTA to neutravidin analyzed with size exclusion chromatography; above an overlay and below a waterfall diagram for better distinction.

To determine a ratio of scFv(AM1)-P-BAP/neutravidin that guarantees free binding sites for radiolabeled biotin a model system was used that reflects the reaction of the biotinylated antibody construct with neutravidin. For this, neutravidin was incubated with different concentration of PEGylated biotin-NOTA (M_w_ ≈ 2300 g/mol) and the amount of biotin bound to neutravidin was analyzed by size exclusion chromatography (SEC). Due to the increase of molecular weight neutravidin/biotin conjugates are expected to show a decreased retention time on the SEC column. However, PEGylation of the biotin derivatives resulted in additional interactions with the column showing higher retention times (Fig. 3).

Therefore, the depicted shoulder signal at higher retention times indicates conjugation of Neutravidin with one single PEG-biotin. At 0.8 eq PEG-Biotin-Bn-NOTA the shoulder signal gets remarkably broader which is related to a conjugation with more than one biotin molecule. Consequently, neutravidin was incubated with only 0.5 eq of biotinylated single chain antibody to ensure free biotin binding sites on the neutravidin-antibody complex.

### Time of maximum cellular accumulation *in vitro*

**Figure 4:**
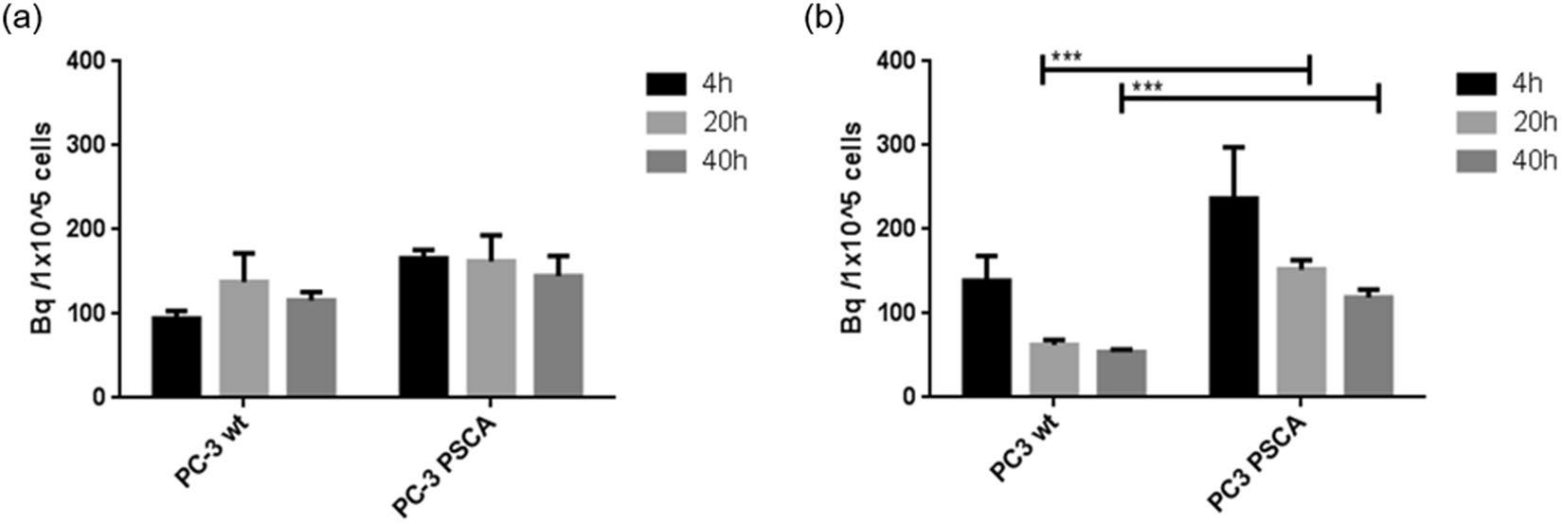
Cellular uptake of scFv(AM1)-P-BAP/neutravidin conjugate and biotin-NODA-GA[^68^Ga] in PC-3 wt and PC-2 PSCA cells after 4, 20 and 40 h pre-incubation of the cells with the scFv(AM1)-P-BAP/neutravidin conjugate. Then radiolabeled biotin was added and incubation continued for (**a**) 1 h or (**b**) 2 h prior to analysis; n = 3 (***p < 0.001; unpaired t-test).

As evaluated by studies with directly ^99m^Tc-labeled scFv(AM1)-P-BAP the scFv shows only marginal internalization which is prerequisite for a pretargeting strategy (Figure S6). To predict binding kinetics of the scFv(AM1)-P-BAP/neutravidin conjugate and the biotin-derivatives the pretargeting strategy was evaluated on human prostate cancer PC-3 wildtype (PC-3 wt, PSCA negative) and PC-3 PSCA cells overexpressing the antigen. Different pre-incubation times with the antibody/neutravidin construct and incubation times with radiolabeled biotin were investigated. As shown by Fig. 4 the single chain antibody scFv(AM1)-P-BAP retains its specificity for PSCA after reaction with neutravidin as the PSCA-positive cells showed a 1.5-fold higher uptake compared to the negative control. Flow cytometric analysis of PC-3 wt and PC-3 PSCA cells further confirmed the antibody specificity for PSCA (SI Figure S5). The uptake in the PC-3 wt (Fig. 4) is probably due to the incorporation of radiolabeled biotin. It is known that fast growing cells like tumor cells have a strong requirement of essential vitamins e.g. biotin^38^. The receptors involved in vitamin internalization are overexpressed on the surface of tumors resulting in a non-specific uptake of radioactivity in the wildtype cells. The incubation with radiolabeled biotin for 2 h resulted in an increased rate of cell-bound radioactivity. Thus, the reaction of neutravidin with biotin required more than one hour. This could be explained by lower affinity of biotin to neutravidin due to the derivatization with the chelator^39^. Considering the binding kinetics of scFv(AM1)-P-BAP in PSCA positive and negative tumor cells, the incubation for 20 h resulted in the highest target/non-target ratio. Nevertheless, a tendency for decreased accumulation with higher incubation times is apparent (Fig. 4a) indicating non-specific, reversible binding.

Based on these *in vitro* binding studies an application of radiolabeled biotin 20–24 h post injection of scFv(AM1)-P-BAP is most promising to achieve the best target/non-target ratio *in vivo*. As shown by the I*n vivo* experiments with the direct labeled scFv(AM1)-P-BAP ([^64^Cu]Cu-NOTA-scFv(AM1)-P-BAP) (SI Figure S7) the peak accumulation of the tracer in the tumor was detected at 24 h post injection defining this time point as optimal for biotin administration.

### Target specificity of scFv(AM1)-P-BAP *in vivo*

**Figure 5:**
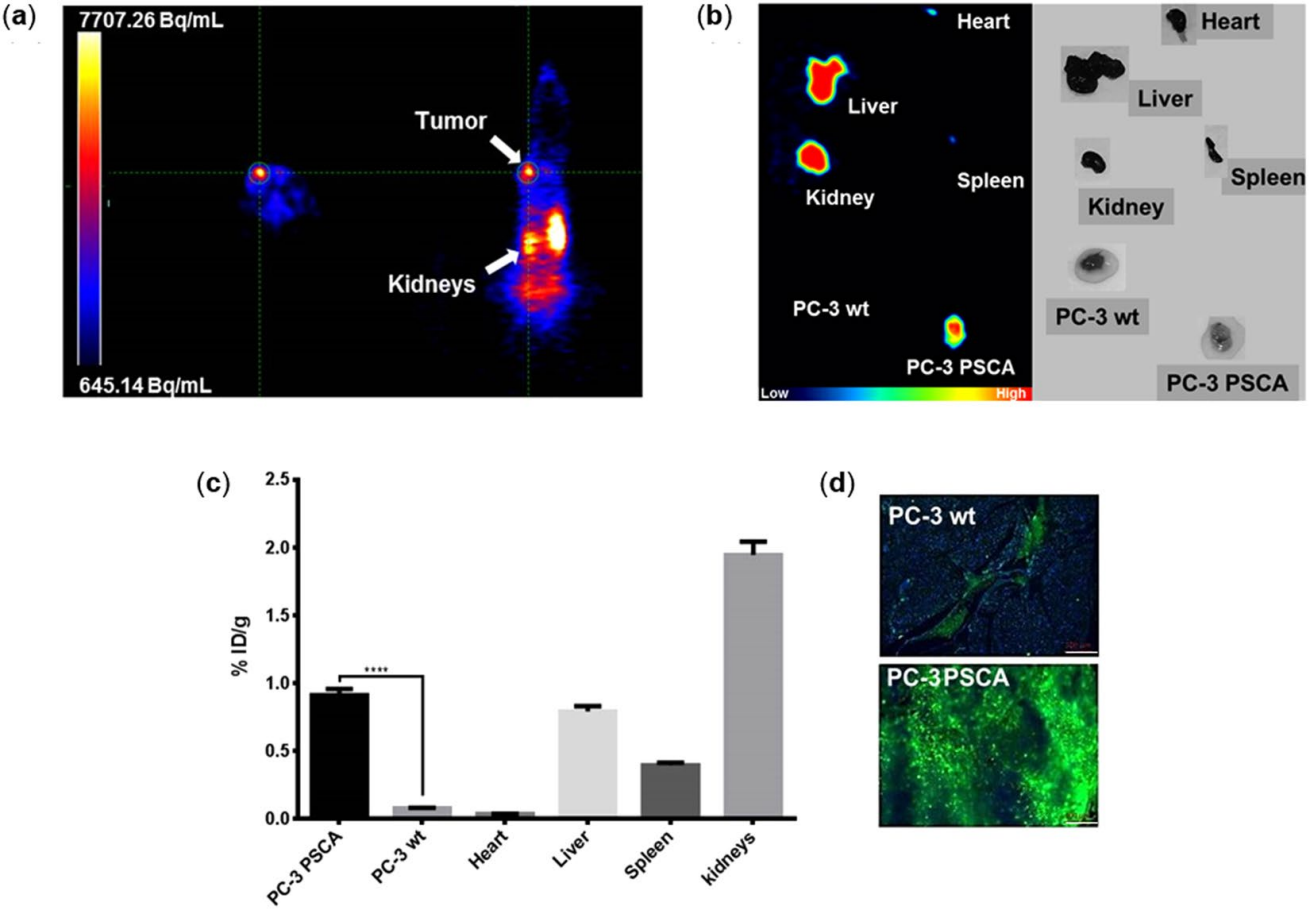
*In vivo* studies in PC-3 wt and PC-3 PSCA xenografted CB17 mice; (**a**) PET-images (left axial, right coronal) of a PC-3 PSCA xenografted mouse after 24 h post injection of the scFv conjugate and 2 h circulation of 6.9 MBq PEG-biotin-NOTA[^68^Ga]; (**b**) *ex vivo* PET-images of a PC3 wt/PSCA xenografted mouse after 24 h post injection of scFv conjugate and 2 h circulation of 13.3 MBq PEG-biotin-NOTA[^68^Ga]; (**c**) the accumulation of radioactivity in percentage of injected dose per gram tissue calculated after measurement with gamma-counter counter (****p < 0.0001; unpaired t-test); (**d**) immunohistochemical analysis of PSCA expression in PC-3 and PC-3 PSCA tumor tissue sections (10 fold magnification).

To prove the specificity of the single chain antibody after conjugation to neutravidin *in vivo* the pretargeting strategy was evaluated in PSCA positive and negative prostate cancer cell xenografted CB17 scid mice. Based on the *in vitro* data (Fig. 4) the small animal PET measurements were performed 24 h after application of the scFv(AM1)-P-BAP/neutravidin and 2 h after injection of the radiolabeled biotin. Specific accumulation of the construct was detected in the PSCA expressing tumor (Fig. 5a) whereas the PC-3 wt tumor showed a very low tumor to background ratio. For more differentiated and well-defined visualization of radioactivity in all organs of interest, additional PET measurements were performed *ex vivo*. To quantify the radioactivity accumulation the extracted organs were analyzed by gamma counting. Besides specific tracer accumulation in the PSCA-positive tumor the *ex vivo* images and the gamma counter analysis verify liver, spleen and kidneys as dose-limiting organs showing a relatively high amount of radioactivity (Fig. 5b,c). Importantly, the PC-3 PSCA tumor shows in average a tenfold higher accumulation of the tracer in comparison to the negative control PC-3 wt tumor.

These data correlate with the expression levels of PSCA determined by immunohistochemical analysis of resected tumor tissue sections (Fig. 5d). The uptake in the kidneys is related to the renal excretion of the unbounded free radiolabeled biotin. However, to decrease the uptake in the liver, which is another dose limiting organ, different biotin derivatives were tested.

### Improved tumor to liver ratio

**Figure 6:**
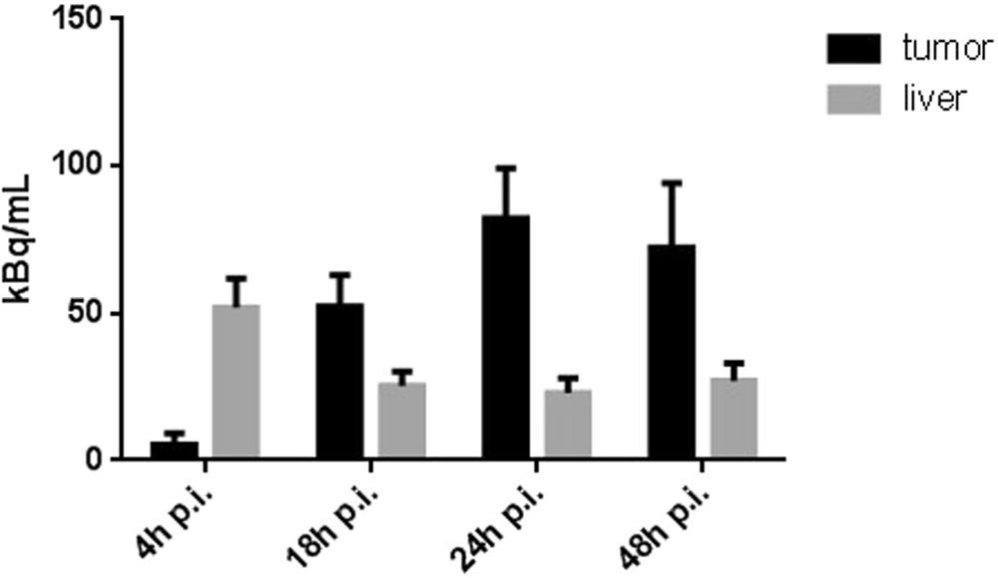
Accumulated radioactivity per volume tissue measured with small animal PET in CB17 xenografted PC-3 PSCA mouse after different incubation times of scFv(AM1)-P-BAP/neutravidin and 6.4 MBq PEG-biotin-DFO[^89^Zr].

The pretargeting strategy was evaluated *in vivo* in a CB 17 scid mouse xenografted with PC-3 PSCA tumor cells in late-stage imaging to determine the point in time of minimal liver accumulation using ^89^Zr-labeled PEG-biotin-DFO. After administration of scFv(AM1)-P-BAP/neutravidin conjugate and its specific tumor accumulation within 24 h, ^89^Zr-labeled PEG-biotin-DFO was injected. The activity in tumor as well as in the liver was determined with a small animal PET after different circulation times of radiolabeled biotin (4–48 h). The accumulation in the liver decreased with increasing circulation time since non targeted macromolecules larger than 50 kDa like the scFv(AM1)-P-BAP/(neutr)avidin conjugate are hepatobiliary excreted^5^. Simultaneously, radioactivity accumulation in the tumor increased which finally resulted in the highest tumor to liver ratio at 24 h post injection of PEG-biotin-DFO[^89^Zr] (Fig. 6). However, keeping in mind the radiation dose deliverd to normal tissue the tumor/liver ratio at erliear time points needs to be optimized. In future studies with the scFv(AM1)-P-BAP this might be achieved by an additional treatment with a clearing agent prior to application of radiolabeled biotin. As shown by Lewis *et al*. for clearing agents like Biotin-GalNAc16 or biotin-N-acetylgalactosamine more than 90% of circulating antibody/streptavidin complex has been removed leading to a higher tumor/liver ratio^40^, and the tracer accumulation in the liver was significantly decreased^41^.

**Figure 7:**
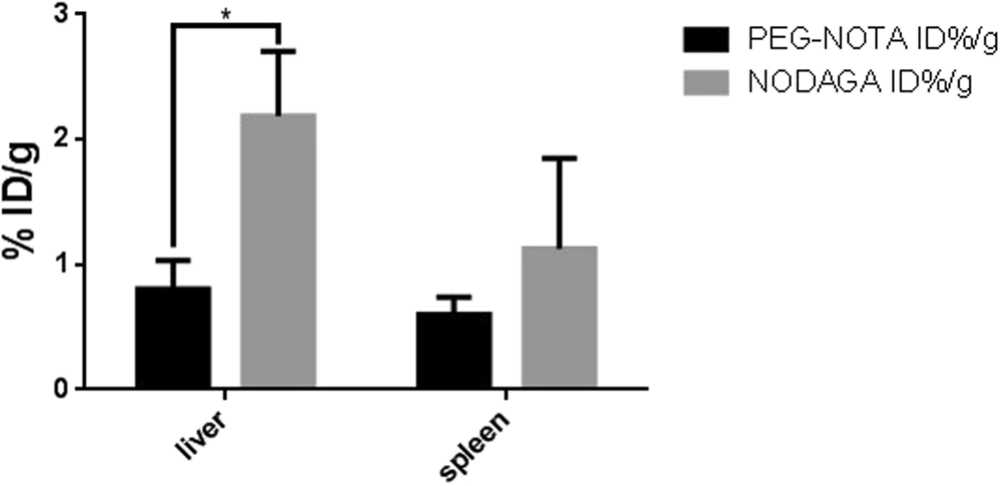
Radioactivity accumulation in the liver and spleen as percent injected dose per gram organ 26 h post injection of two different biotin derivatives. The mice were euthanized and the organs of interest were harvested. The measurement of the radioactivity in the different organs was performed with gamma counting. (n = 4, *p < 0.05, unpaired t-test).

Furthermore, the impact of a PEG (n = 45) linker on the tumor to liver ratio of biotin was investigated (Fig. 7). The tumor data showed no significant difference between the PEGylated and the non-PEGylated conjugate. However, the non-PEGylated conjugate showed a high accumulation in liver and spleen while this accumulation effect was considerably decreased in these dose-limiting organs when the tracer was conjugated with a PEG linker.

The accumulation in liver and spleen can be explained by non-specific uptake of the conjugates through cells of the mononuclear phagocyte system particularly by Kupffer cells and splenocytes.

Regarding the retention in circulation of the two different biotin derivatives, the tumor to heart ratio is also not significantly changed (SI Figure S8). This indicates that a prolonged circulation due to PEGylation is not responsible for the different amount of accumulation detected in liver. Therefore, the significantly reduced accumulation of the PEGylated biotin in the liver is more likely related to a lower uptake by Kupffer cells^42^. This results in a favored biodistribution for future diagnostic and therapeutic applications.

## Conclusion

In this study, we present a pretargeting strategy with the scFv(AM1)-P-BAP/Neutravidin conjugate and its potential for imaging PSCA expressing tumor cells *in vitro* and *in vivo*. Three biotin/chelator conjugates which differ by their conjugation chemistry, the linker and the chelator were synthesized. *In vitro* studies prove a specific binding to a PSCA-positive cell line with a 1.5-fold higher uptake compared to the negative control. The specific binding was confirmed in *in vivo* studies in which PSCA-positive tumor xenografts exhibit a tenfold higher tracer accumulation compared to the control PC-3 wt tumors. PEGylation of radiolabeled biotin significantly reduced uptake in the liver. Thus, the pretargeting strategy presented in this study is a promising approach for addressing PSCA expressing tumors. Further optimization of the administration schedule with an additional cleaning reagent treatment will further improve this approach towards a theranostic use of scFv(AM1)-P-BAP against PSCA expressing tumors.

## Methods

### Synthesis of biotin/chelator conjugates

All biotin/chelator conjugates were synthesized at room temperature at a pH of 9.0–9.5 in aqueous solution. In a typical reaction 1.0 eq. of biotin derivative was reacted with 1.0–1.5 eq. of chelator. All products were purified by reversed phase radio-HPLC (C18 column, flow rate: 1 mL/min) (For further details see SI). All conjugates were characterized with reversed phase radio-HPLC (Knauer Smartline Pump 1000, C18 column) and ESI-MS (Finnigan SSQ 7000).

### Radiolabeling

^68^Ga-labeling: To label the NOTA conjugates radioactively a 3 M NH_4_OAc solution (1/10 of the volume of the used [^68^Ga]GaCl_3_ solution) was added to the reaction mixture. Afterwards n.c.a. [^68^Ga]GaCl_3_ (10–20 MBq) was added to the NOTA conjugate. The reactions were analyzed with reversed phase radio-HPLC and radio-TLC (citrate buffer with a pH = 5.5 as mobile phase, silica acid as stationary phase).

^89^Zr-labeling: For labeling PEG-biotin-DFO 1 M Na_2_CO_3_ solution was added to [^89^Zr]Zr(C_2_O_4_)_2_ (10 MBq, Perkin Elmar) in a volume ratio of 1:1. Afterwards the biotin-DFO conjugate was added and the reaction mixture was diluted with PBS buffer (pH = 7.4). The reaction took place at room temperature at pH ≈ 7 for 1–2 h. Reaction control of radioactive labeling was performed with radio-TLC (5 mM DTPA at pH = 8–9 as mobile phase, silica acid as stationary phase).

### Reaction of biotinylated scFv(AM1)-P-BAP with neutravidin

The generation of biotinylated scFv(AM1)-P-BAP was accomplished as described recently^37^. 1.0 eq. of neutravidin reacted with 0.5 eq. single chain antibody. The reaction took place at room temperature and was analyzed with size exclusion chromatography (0.5% SDS as mobile phase).

### *In vitro* experiments

*In vitro* studies were performed with a human prostate cancer cell line (PC-3). PC-3 PSCA^43^ as well as PC-3 wt cells were seeded on 12 well plates and kept under standardized cell culture conditions (37 °C, 5% CO_2_). To investigate the pretargeting strategy the scFv(AM1)-P-BAP/neutravidin conjugate (0.083 nmol (6.56 µg)/0.166 nmol (9.96 µg/well)) was incubated for defined time with each cell line. ^68^Ga-labeled biotin (0.5–1.5 MBq per well) was added and incubated for another 1 or 2 h under standardized cell culture conditions. Afterwards the cells were harvested and radioactivity was measured in the collected medium and in the cells with a gamma-counter (*Perkin Elmar*, Wizard23). The cellular uptake was normalized to the cell number.

### *In vivo* experiments

For evaluation of the general kinetic and the pretargeting strategy *in vivo* CB 17 scid mice were subcutaneously injected with PC-3 wt and PC-3 PSCA cells (1 × 10^6^ cells per mouse either in flank or neck). The scFv(AM1)-P-BAP/neutravidin (75 µg/120 µg) conjugate was injected intravenously into the tail vein of each mouse. 4 to 24 h post injection the radioactive labeled biotin derivative (5–15 MBq) was injected intraperitoneal. Two hours post biotin injection small animal PET-measurements (*Siemens Inveon*) were performed. All measurements were performed dynamically for 60 min followed by 10 min transmission. After data acquisition, PET images were reconstructed by a 3-dimensional ordered-subsets expectation maximum (OSEM) algorithm. All data were corrected for attenuation, scatter, dead time and decay. For calculation of organ accumulated radioactivity during the analyzed time period (time activity curve, TAC), regions of interest were drawn over the major organs and tumor on the whole-body images. The mice were euthanized 3–4 h post biotin injection and organs of interest were harvested. To evaluate the radioactivity concentration, the organs were wet weighted and the accumulated radioactivity was measured with gamma counter (*PerkinElmer Inc*.). The decay-corrected radioactivity was expressed as percentage of injected dose per gram (% ID, mean ± SD) which was obtained by dividing tissue radioactivity by injected dose assuming the tissue density as 1 g/cm^3^. The number of animals per group was calculated via a power calculation using G*Power, Version 3.1.9.2 (*Freeware*, *Kiel University*, *Kiel*, *Germany*). Results are expressed as Mean +/− SD. All statistical calculations were performed using Graph Pad Prism version 6.00 for Windows. Unpaired t-test with post-hoc comparisons was performed with Welch´s correction. Effects were considered to be statistically significant if p ≤ 0.05.

### Data availability statement

The datasets generated during and/or analyzed during the current study are available from the corresponding author on reasonable request.

### Ethical statement

Animal experiments were performed in accordance with the German legislation governing animal studies following the ‘Guide for the care and use of Laboratory Animals’ (NIH publication, 8th edition, 2011) and the Directive 2010/63/EU on the protection of animals used for scientific purposes (Official Journal of the European Union, 2010). Official permission was granted from the governmental animal care and use office (LANUV Nordrhein-Westfalen, Recklinghausen, Germany).

## Electronic supplementary material

Supporting Information:
