# Supplementary material for: Evaluation of a Pretargeting Strategy for Molecular Imaging of the Prostate Stem Cell Antigen with a Single Chain Antibody
Source: Sci Rep. 2018 Feb 28;8:3755. doi: 10.1038/s41598-018-22179-y (PMC5830539; doi:10.1038/s41598-018-22179-y)
Supplement: Supplementary file 1 — Supporting Information: [file 41598_2018_22179_MOESM1_ESM.docx]

**Supporting Information: Evaluation of a Pretargeting Strategy for Molecular Imaging of the Prostate Stem Cell Antigen with a Single Chain Antibody**

Lena Tienken^1,*^, Natascha Drude^1*^, Isabell Schau^2^, Oliver H. Winz^1^, Achim Temme^2, 3^, Elmar Weinhold^4^, Felix M. Mottaghy^1, 5^, Agnieszka Morgenroth^1^

1 Department of Nuclear Medicine, University Hospital RWTH Aachen, 52074 Aachen, Germany

2 Department of Neurosurgery, Experimental Neurosurgery/ Tumor Immunology, TU Dresden, 01307 Dresden, Germany

3 German Cancer Consortium (DKTK), partner site Dresden; German Cancer Research Center (DKFZ), Heidelberg, Germany

4 Institute of Organic Chemistry, RWTH Aachen University, 52074 Aachen, Germany

5 Department of Radiology and Nuclear Medicine, MUMC+, Maastricht, The Netherlands

*contributed equally

**Contents:**

1. Schematic presentation of the scFv(AM1)-P-BAP construct
2. Experimental procedures
   1. Synthesis and characterization of the biotin/chelator conjugates
   2. Radiolabeling of AM1 with [^99m^Tc]
   3. Internalization study with [^99m^Tc] scFv(AM1)-P-BAP
3. Additional results
   1. FACS analysis of PC-3 wt and PC-3 PSCA cell lines
   2. Internalization behavior of scFv(AM1)-P-BAP
   3. Biodistribution of the direct labeled scFv(AM1)-P-BAP

3.4 Tumor-to-heart ratio – comparison of PEGylated and non-PEGylated biotin conjugates

1. References
2. **Schematic presentation of the scFv(AM1)-P-BAP construct**


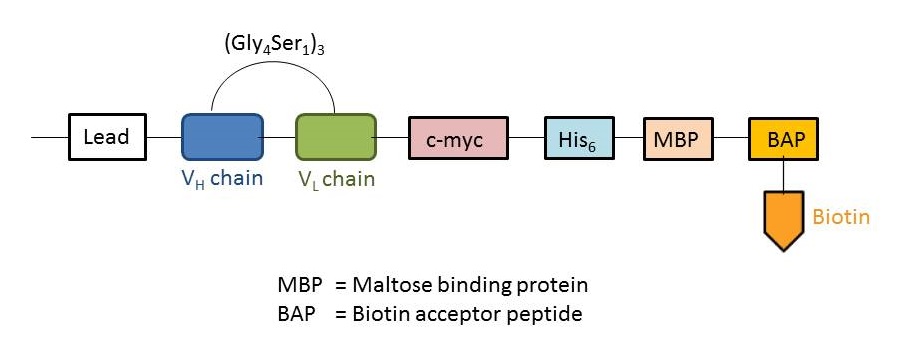


*Figure S1: Scheme of the structure of the scFv(AM1)-P-BAP construct.*

A summarized scheme of the scFv(AM1)-P-BAP is shown to demonstrate the structure and the recognition site for biotin (BAP) or rather neutravidin.

1. **Experimental procedures**

2.1 Synthesis and characterization of the biotin/chelator conjugates:


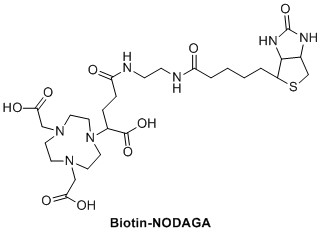


Biotin-NODAGA without PEG linker was prepared by adding 1.0 eq (+)-biotin-N-hydroxysuccinimide-ester (2.05 mg, 6.00 µmol, Thermo Scientific) and 2.0 eq triethylamine (1.21 mg, 12.0 µmol) dissolved in 200µL metal-free H_2_O to 1.0 eq NH_2_-NODAGA (2.50 mg, 6.00 µmol, Macrocylcics) in 800 µL dimethylacetamide (DMA, Merck). The pH of the solution was 9.0-9.5. Full conversion of the starting materials to product was achieved at room temperature overnight. DMA was removed under reduced pressure and the product was diluted and stored in metal-free H_2_O at -20°C. Analysis of the product (ESI-MS (positive mode) m/z: 644.4 [M^+^H]^+^) after radiolabeling with 68Ga by reversed phase HPLC yielded a purity of >95% (Figure S2).


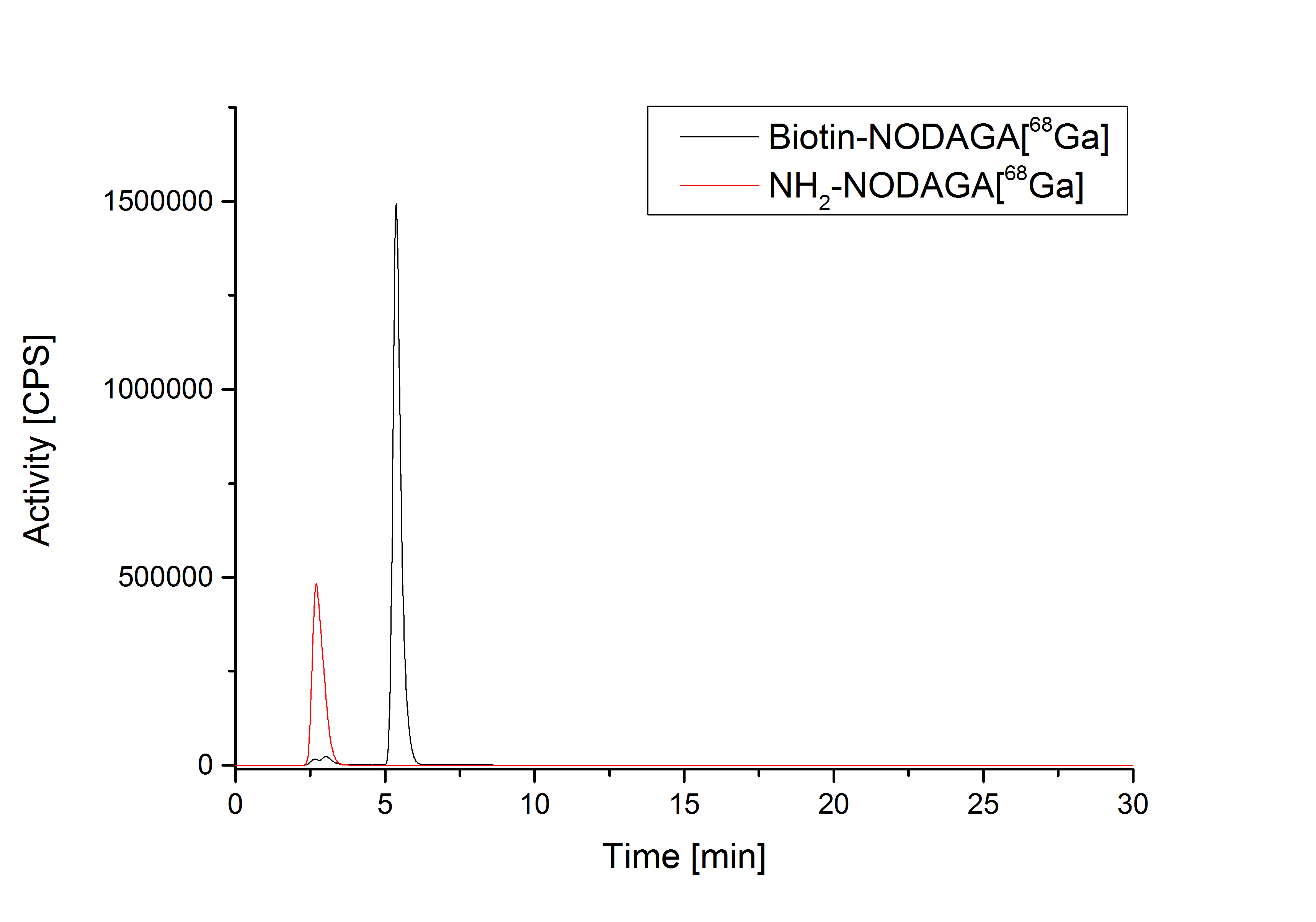

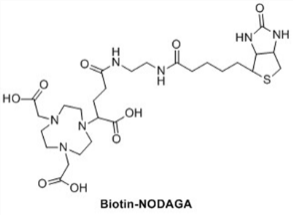


*Figure S2: HPLC chromatograms of purified Biotin-NODAGA and as comparison the unbound chelator after radioactive labeling with ^68^Ga.*


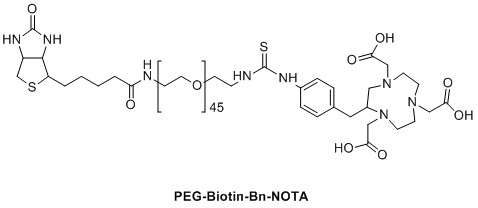


For the PEGylated biotin-NOTA conjugate a solution of 1.0 eq polyethylenglykol-2-amino-ethylether-biotin (3.45 mg, 1.5 µmol, Thermo Scientific) in 500 µL PBS buffer (pH= 7.4, PAN Biotech) was prepared. The pH was determined and if necessary adjusted to a value of 9.0 with a 0.1 M Na_2_CO_3_ solution (1.0 µL). 1.5 Eq of the chelator p-SCN-Bn-NOTA (1.26 mg, 2.25 µmol, Macrocylics) were dissolved in 300 µL PBS buffer (pH= 7.4, PAN Biotech) and added to the first solution. The pH was measured again and adjusted to a value of 9.0. The reaction was performed at room temperature overnight. The product was freeze-dried and stored in metal-free H_2_O at -20°C. Analysis of the product by reversed phase HPLC after radiolabeling with ^68^Ga indicates a purity of >85% (Figure S3).

*Figure S3: Above: HPLC chromatograms of purified PEG-Biotin-Bn-NOTA and as comparison the unbound chelator after radioactive labeling with ^68^Ga.Below: TLC chromatograms (back: reaction mixture after purification with reversed phase HPLC, front: radioactive labeled chelator); The blue circled signal belongs to PEG-Biotin-Bn-NOTA[^68^Ga] as it stays on the start point, the green signal is related to SCN-Bn-NOTA[^68^Ga].*


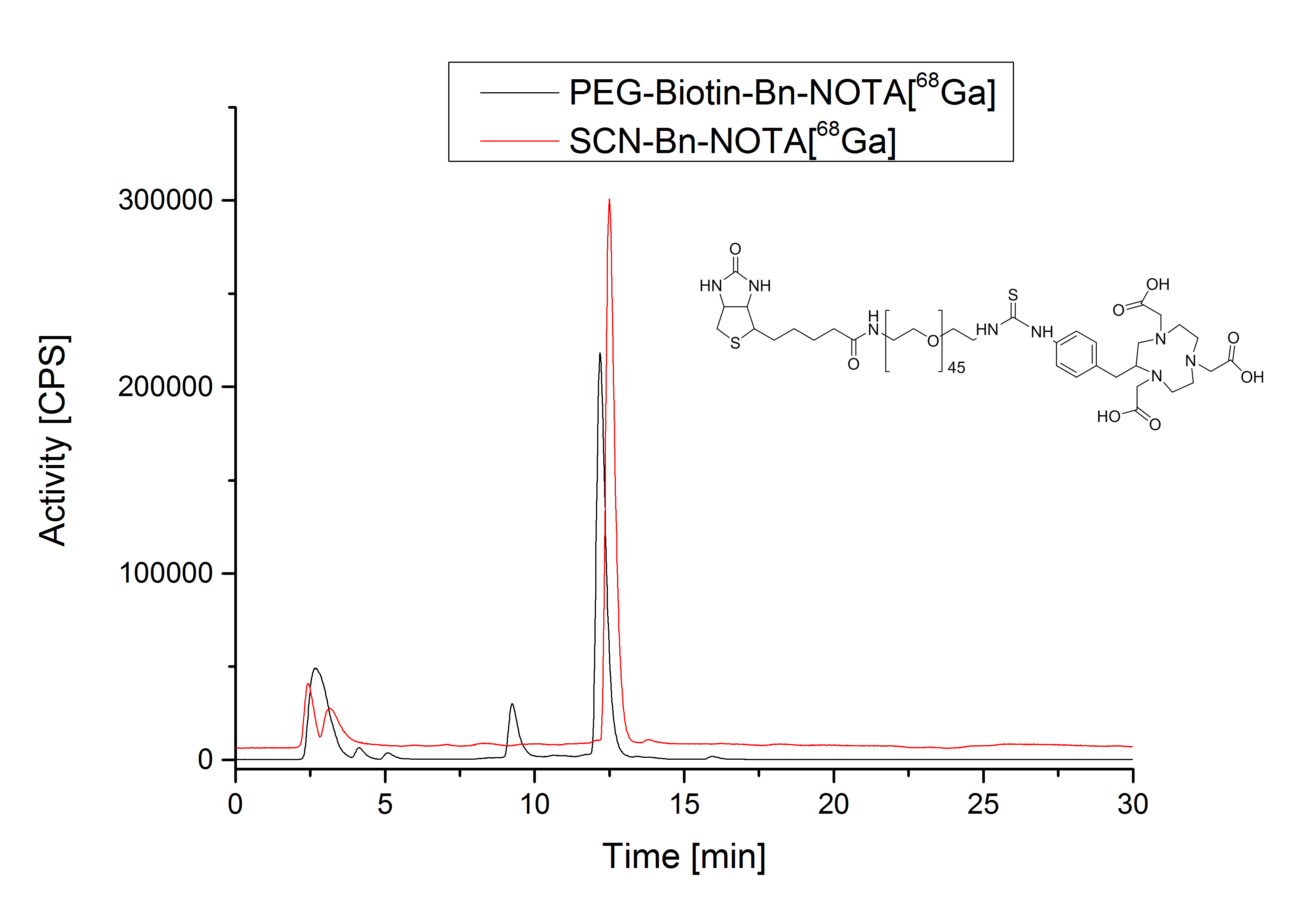


Unbound ^68^GaCl_3_


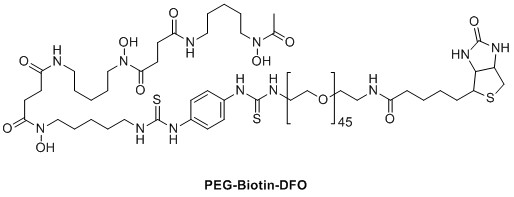


For the synthesis of PEG-Biotin-Bn-DFO polyethylenglycol-2-amino-ethylether-biotin (3.45 mg, 1.50 µmol, 1.0 eq) was dissolved in 200 µL PBS buffer (pH= 7.4, PAN Biotech). The chelator p-SCN-deferoxamine (1.51 mg, 2.00 µmol, 1.3 eq) was dissolved in 100 µL DMSO (Merck) and added to the first solution. The pH value was determined (pH= 9.0-9.5). The reaction was performed at room temperature overnight. The product was purified by reversed phase HPLC, freeze-dried and dissolved in 500 µL metal-free H_2_O. Analysis of the product by reverse-phase HPLC after radiolabeling with ^68^Ga indicates a purity of >90% (Figure S4).


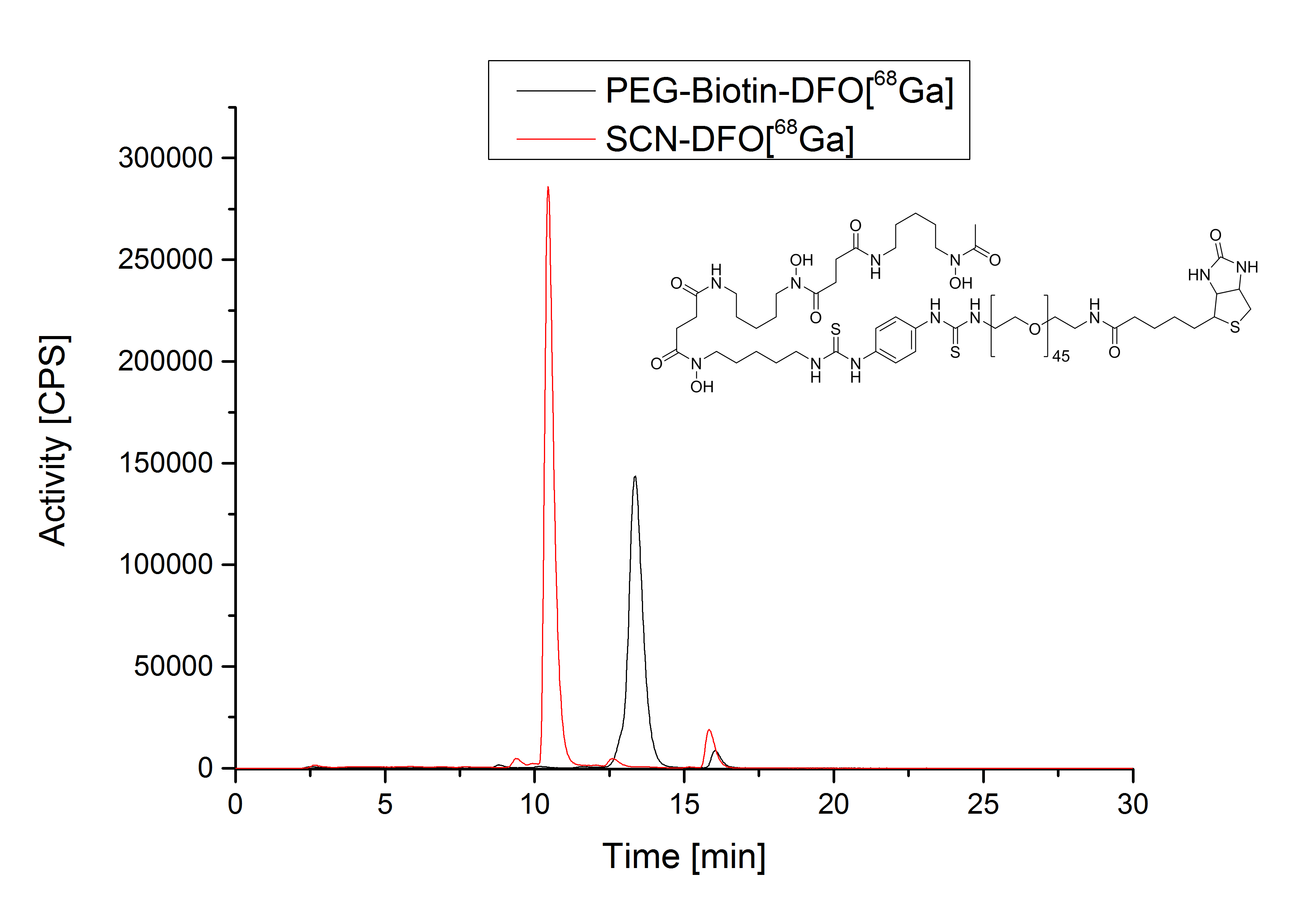


*Figure S4: HPLC chromatograms of purified PEG-Biotin-DFO and as comparison the unbound chelator after radioactive labeling with ^68^Ga.*

All products were purified after radiolabeling with n.c.a. ^68^Ga by reversed phase radioHPLC (Knauer Smartline Pump 1000, C18 column). Compounds were eluted with acetonitrile (Merck) (10% for 20 min, 10–75% in 5 min) in aqueous 0.1% TFA with a flow of 1 mL/min.

- 1. Radiolabeling of scFv(AM1)-P-BAP with [^99m^Tc]

The scFv AM1 antibody was directly labelled via its His tag^[1]^ with [^99m^Tc]technetium tricarbonyl by the following procedure: the ^99m^Tc(I) tricarbonyl compound [^99m^Tc(H_2_O)_3_(CO)_3_]^+^ was generated using a commercial kit "Isolink" from Mallinckrodt Nuclear and [^99m^Tc]pertechnetate from a clinical generator (also from Mallinckrodt Nuclear, now: Curium). Briefly, 0.9 mL [^99m^Tc]pertechnetate in saline (435 MBq) were added to the kit which was then let reacted at 100°C for 20 min. The radiochemical purity of the tricarbonyl was > 99%. 0.5 mL of that solution were neutralized by addition of 60 µL 1 M H_3_PO_4_ and added to 12.02 µg scFv(AM1)-P-BAP (240.33 ng/µL). The vial was sealed and continuously aerated with nitrogen at a temperature of 39°C. The reaction proceeded for 4 h. When the volume in this period undercut 100 µL, 200 µL of sterile water were added. The solution was transferred onto a filter unit with 10 kDa cutoff and was centrifuged at 13.000 rpm for 10 min. The labelled protein was washed with 400 µL PBS and again centrifuged. The residual protein solution of 50 µL was transferred to a sample tube, the filter washed with 100 µL PBS and united with the protein solution (150 µL in total, 31 MBq) which was further centrifuged for 10 min at 13.000 rpm. The supernatant was used in cell uptake experiments.

- 1. Internalization study with [^99m^Tc]AM1

For internalization study the PC-3PSCA cells were seeded (5*10^4 cells/well, 12-well plate) 48h prior to incubation with the tracer. After wash step with fresh medium the cells have been incubated with [^99m^Tc]-labeled scFv(AM1)-P-BAP (200kBq/well) at 37°C and 5% CO_2_. After 1h, 4h and 24h the binding has been stopped by washing the cells with ice cold PBS. To obtain the membrane bonded tracer, the cells have been washed twice with acidic glycine buffer (0.06 mol/L glycine, 150 mmol/L NaCl, pH 2.8). To quantify the internalized fraction of scFv(AM1)-P-BAP, the cells have been treated for 10 min @ 37°C with NaOH (1M). Collected fractions have been measured by gamma counter, and the cell membrane bounded and internalized fraction were calculated as % of injected dose (ID) per well.

1. **Additional results**

3.1 FACS analysis of PC-3 wt and PC-3 PSCA cell lines

The expression level of PSCA on the PC-3 wt and PC-3 PSCA cell lines were determined by flow cytometry (FACS). For this, the cells were incubated with the parental antibody anti-PSCA-Ab 7F5 (a-PSCA) and with an IgG antibody unspecific for PSCA (a-mouse IgG) as control. The antibodies were labeled with the fluorophore Alexa Fluor 555. As negative control cells were measured without any pretreatment.


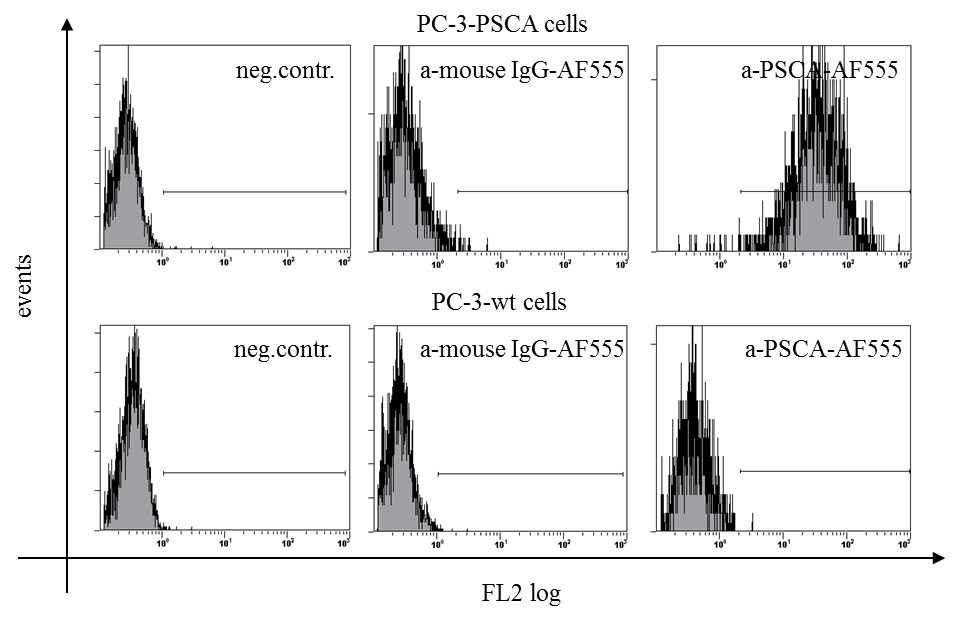


*Figure S5: FACS analysis of the PC-3 cell lines. After accumulation of the parental anti-PSCA antibody it is fluorescence labeled with α-m-AF 555 so that the PSCA expression can be determined.*

The FACS data shows that the incubation with the parental anti-PSCA antibody leads to an uptake of 100% in the PC-3 PSCA cells whereas no uptake is measured with the PSCA-unspecific a-mouse IgG antibody (Figure S5 above) proving that the binding is PSCA-specific. The PC-3 wt cells show no uptake neither with the anti-PSCA nor with the anti-mouse antibody. Therefore, the PC-3 wt cells are PSCA-negative.

- 1. Internalization kinetics of scFv(AM1)-P-BAP

Internalization kinetics of surface-bound [^99m^Tc] were measured on PC-3 PSCA cells (Figure S6). Using the directly radiolabeled scFv(AM1)-P-BAPand quantification of samples the time at we identified a slow internalization of scFv(AM1)-P-BAP. After 4h more than 75% of bonded scFv(AM1)-P-BAP was localized ion the cell surface, while incubation for additional 20h led to an internalization rate of about 60% of scFv(AM1)-P-BAP.

*Figure S6: Internalization study with [^99m^Tc]*scFv(AM1)-P-BAP *and PC-3 PSCA cells. After incubation with 200kBq per well the cells were treated with acidic glycine buffer and NAOH to obtain the cell surfaces bonded and internalized fractions of scFv(AM1)-P-BAP, respectively. The amount of cell retained scFv(AM1)-P-BAP fractions was quantified by gamma counting (n=3; P < 0.001).*

- 1. Biodistribution of the direct labeled scFv(AM1)-P-BAP

The scFv(AM1)-P-BAP was directly bound to chelator SCN-NOTA via the N-term of the peptide. This construct was radioactive labeled with the positron emitter ^64^Cu (half-life t_1/2_= 12.7h) to visualize the time-dependent biodistribution using PET imaging. The PET images as well as the calculated radioactivity in the tumor (by analysis of region of interests (ROIs)) defines 24h post injection of the scFv(AM1)-P-BAP as point in time with highest tumor accumulation (Figure S7) and consequently, as the appropriate moment for biotin injection during the evaluated pretargeting strategy.


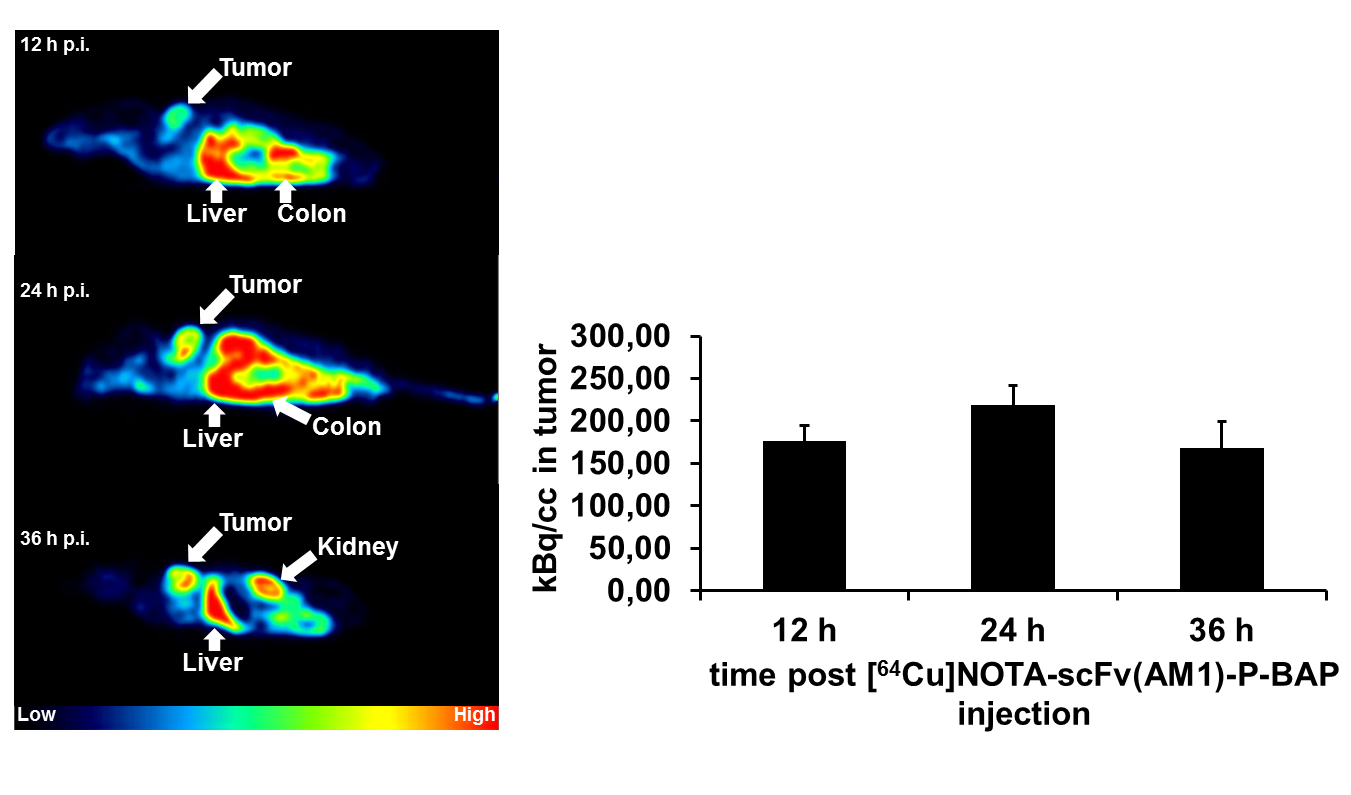


*Figure S7: Biodistribution of the direct labeled scFv(AM1)-P-BAP to evaluate the point in time of maximum tumor accumulation of the antibody construct; left: PET images at different points in time, right: calculated tumor accumulation.*

3.4 Tumor-to-heart ratio – comparison of PEGylated and non-PEGylated biotin conjugates:

After 24h circulation time of the scFv(AM1)-P-BAP/neutravidin conjugate and 2h post injection of different biotin conjugates the relevant organs were removed and measured with gamma-counter (Wizard^2^3, Perkin Elmer). The tumor-to-heart ratios were calculated (Graph Pad Prism Version 6.00, San Diego California USA) to determine whether PEGylation results in a prolonged biological half-life.


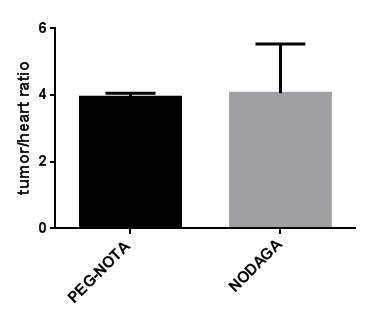


*Figure S8: Tumor-to-heart ratio of the PEGylated and the non-PEGylated biotin conjugates in resected organs.*

On the contrary to the described prolonging effects of PEGylation on the serum half-life^[2]^ the calculated tumor-to-heart ratios show no significant difference between the PEGylated and non-PEGylated biotin which indicates that PEGylation does not have an impact on retention in blood circulation (Figure S8).

1. **References**

[1] Waibel, R.; Alberto, R.; Willuda, J.; Finnern, R.; Schibli, R.; Stichelberger, A.; Egli, A.; Abram, U.; Mach, J.P.; Plückthun, A.; Schubiger, P.A. Stable one-step technetium-99m labeling of His-tagged recombinant proteins with a novel Tc(I)-carbonyl complex. Nat Biotechnol. 1999, 17, 897-901.

[2] Yang, K.; Basu, A.; Wang, M.; Chintala, R.; Hsieh, M.-C.; Liu, S.; Hua, J.; Zhang, Z.; Zhou, J.; Li, M.; Phyu, H.; Petti, G.; Mendez, M.; Janjua, H.; Peng, P.; Longley, C.; Borowski, V.; Mehlig, M.; Filpula, D., Tailoring structure-function and pharmaconkinetic properties of single-chain Fv proteins by site-specific PEGylation. Protein Engineering Design and Selection 2003, 16, 761-770.
